# The degenerome—a novel streamline-wise approach for white matter integrity in neurodegeneration

**DOI:** 10.1038/s41531-026-01428-2

**Published:** 2026-06-10

**Authors:** Jonas A. Hosp, Marco Reisert, Nils Schröter, Elias Kellner, Hansjörg Mast, Michel Rijntjes, Sabine Hellwig, Lars Frings, Heinz Wiendl, Horst Urbach, Alexander Rau

**Affiliations:** 1 Department of Neurology and Clinical Neuroscience, Medical Center, Faculty of Medicine, University of Freiburg, Freiburg, Germany; 2 Department of Stereotactic and Functional Neurosurgery, Medical Center, Faculty of Medicine, University of Freiburg, Freiburg, Germany; 3 Medical Physics, Department of Diagnostic and Interventional Radiology, Medical Center, Faculty of Medicine, University of Freiburg, Freiburg, Germany; 4 Department of Neuroradiology, Medical Center, Faculty of Medicine, University of Freiburg, Freiburg, Germany; 5 Department of Psychiatry, Medical Center, Faculty of Medicine, University of Freiburg, Freiburg, Germany; 6 Department of Nuclear Medicine, Medical Center, Faculty of Medicine, University of Freiburg, Freiburg, Germany

**Keywords:** Biomarkers, Neurology, Neuroscience

## Abstract

Neurodegenerative diseases impair both gray matter and long-range white-matter pathways. Existing diffusion MRI approaches are either local or depend on predefined parcellations, limiting their ability to capture distributed network disruption. We introduce a streamline-wise framework to map axonal degeneration and derive disease-specific fiber “degeneromes” across Alzheimer’s disease (AD), Parkinson’s disease (PD), multiple system atrophy (MSA), and progressive supranuclear palsy (PSP). We analyzed diffusion microstructure imaging and T1-weighted MRI (3 T Siemens Prisma, 2018–2024) in AD (*n* = 81), PD (*n* = 177), MSA (*n* = 50), PSP (*n* = 35), and healthy controls (*n* = 26). The intraaxonal volume fraction, estimated via a Bayesian three-compartment model, was mapped along ~20,000 normative streamlines in MNI space using age- and sex-adjusted regression with FDR correction. Streamline-wise mapping revealed disease-specific degeneromes consistent with established pathoanatomical models: AD involved limbic and temporo-occipital pathways, PD showed commissural and posterior association involvement, MSA affected pontocerebellar and corticospinal tracts, and PSP involved the dentato-rubro-thalamic tract and superior cerebellar peduncle. These signatures supported group-level differentiation, and streamline-wise z-scores enabled intuitive single-patient visualization. Fiber degeneromes offer a connectome-informed biomarker with strong biological plausibility, discriminatory potential across neurodegenerative entities, and a clear route toward clinical single-patient reporting.

## Introduction

Neuronal cell death is a defining hallmark of neurodegenerative diseases and a major driver of clinical progression. Neuronal degeneration and loss manifest as measurable reductions in gray-matter volume, a characteristic feature of Alzheimer’s disease (AD) with mixed amyloid and 3R/4R tau pathology, α-synucleinopathies such as Parkinson’s disease (PD) and multiple system atrophy (MSA) and 4R tauopathies including progressive supranuclear palsy (PSP)^1–6^. Neurodegenerative diseases disrupt not only neuronal cell bodies but also long-range white matter pathways that support distributed brain networks^7–12^. MRI-based biomarker work has traditionally emphasized gray matter atrophy, yet diffusion MRI provides complementary information about tissue injury within white matter^13,14^. Because axonal damage can disconnect distant cortical and subcortical regions, a pathway-aware description of degeneration may capture clinically relevant network effects that are not fully represented by regional gray matter measures alone.

Loss of white-matter integrity can be assessed noninvasively with MRI, and microstructurally informed techniques such as diffusion-weighted imaging offer sensitive markers of neurodegeneration^15,16^. However, commonly used analytic strategies remain limited in scope: voxel- or fixel-based approaches localize effects to very small spatial scales^17^, whereas atlas-based methods are constrained by predefined parcellations^18^. Because white matter is organized into coherent fiber bundles and long-range axonal tracts, a more biologically grounded strategy is to quantify degeneration along pathways themselves. Normative connectomes, rather than static structural templates, can represent white-matter architecture in a tract- and streamline-specific manner and enable statistical inference that follows the trajectories of axonal pathways^19,20^. Earlier tract-aware methods, such as PASTA and AFQ, quantified microstructural variation at points or nodes along selected reconstructed bundles^21–23^, whereas connectometry and bundle-analytic frameworks extended tract-informed inference to local connectome segments or bundle representations across populations^20,24^. We propose an approach that relies on a fixed normative whole-brain streamline scaffold in MNI space to sample subject-specific quantitative MRI maps across the entire connectome without requiring subject-specific tractography, bundle dissection, or cross-subject streamline matching. This outputs a streamline-wise statistical map in common space. Here, we introduce a streamline-wise approach to quantify loss of white-matter integrity across multiple neurodegenerative diseases. This method enables identification of disease-specific patterns of axonal pathway involvement and the definition of entity-associated “degeneromes”.

## Results

### Voxel- and streamline-wise results of white matter microstructure

**Fig. 1:**
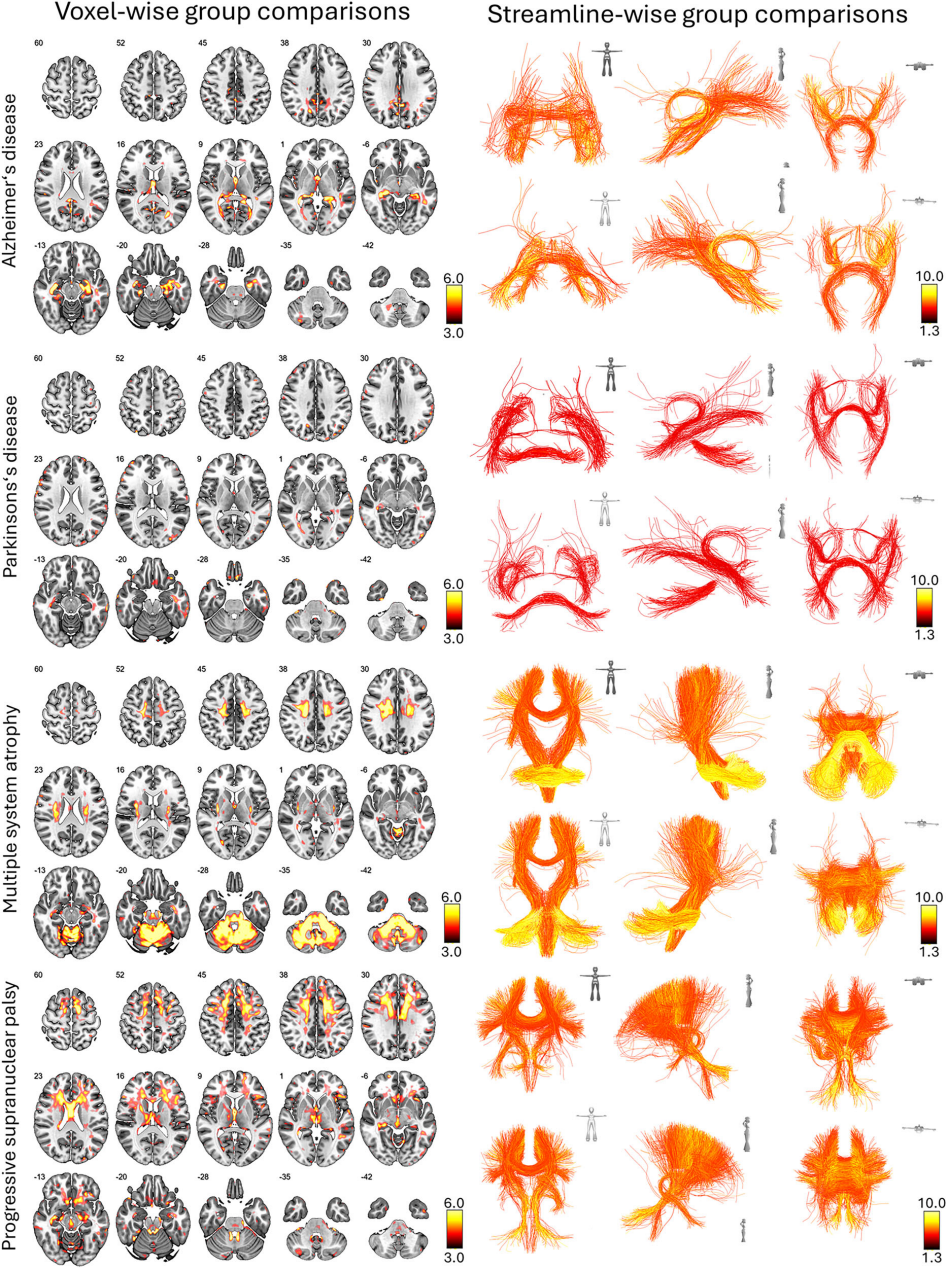
Comparison of voxel-wise group (left column) and streamline-wise group (right column) of the diffusion microstructure imaging-derived intraaxonal volume fraction (V-intra), revealing disease-specific patterns across multiple neurodegenerative disorders in comparison with healthy controls after false discovery rate-correction. Color bars indicate p-values on a log scale.

Significant results of voxel- and streamline-wise group comparisons of V-intra for each diagnostic group (AD, PD, PSP, and MSA) versus healthy controls are shown in Fig. 1.

Voxel-wise whole-brain comparisons of V-intra maps between AD patients and controls revealed an AD-typical pattern characterized by temporomesial and parietal alterations, predominantly affecting gray matter. Whole-connectome, streamline-wise analyses showed primary involvement of the limbic system and temporo-occipital pathways. With reference to the BCBtoolkit white-matter ROIs, the highest overlap was observed in the posterior cingulum (right and left) and the fornix, followed by the optic radiations (right and left), the inferior longitudinal fasciculi (right and left), the anterior commissure, the cingulum (right), and the right uncinate fasciculus (see Fig. 1 and Supplementary Fig. 1 for detailed results).

Voxel-wise comparisons of V-intra maps between PD patients and controls revealed predominantly gray-matter alterations involving the temporomesial and orbitofrontal regions, extending into the occipitoparietal cortex. This pattern was corroborated by streamline-wise analyses, which indicated involvement of the limbic system, long temporo-occipital pathways, and crossing ponto-cerebellar fibers. With reference to the BCBtoolkit white-matter ROIs, the highest overlap was observed in the fornix, followed by the anterior commissure, the optic radiations (right and left), the inferior longitudinal fasciculi (left and right), the uncinate fasciculi (left and right), and the inferior fronto-occipital fasciculi (left and right; see Fig. 1 and Supplementary Fig. 1 for detailed results).

As a result of voxel-based group comparisons of V-intra maps between MSA patients and controls, we observed widespread reduced V-intra in the basal ganglia, pontine and cerebellar parenchyma, as well as in the frontal motoric and premotoric white matter. Streamlines exhibiting significant group differences after FDR correction were found in the pontocerebellar system and long-range motor pathways and especially the corticospinal tract. With reference to the BCBtoolkit white-matter ROIs, the highest overlap was observed in the paracentral U-tracts (right and left) and the corticospinal tracts (right and left), followed by the pons (right and left), the right hands-up U-tract, the right frontal superior longitudinal fasciculus, the right superior longitudinal fasciculus I, and the right fronto-insular tract (see Fig. 1 and Supplementary Fig. 1 for detailed results).

In PSP, voxel-based analyses of V-intra maps with controls showed widespread affection of the frontal white matter, medial thalamus and the midbrain extending to the superior cerebellar peduncles. The streamline-wise approach showed an involvement of the frontal association and commissural fibers with emphasis on the premotor zone, frontal association and commissural fibers, and the dentato-rubro-thalamic tract. With reference to the BCBtoolkit white-matter ROIs, the highest overlap was observed in the frontal superior longitudinal pathways (left and right) and the frontal aslant tracts (right and left), followed by frontal commissural fibers, the right and left frontal inferior longitudinal tracts, the left inferior longitudinal fasciculus, the right fronto-insular tract 4, and the right anterior thalamic projections (see Fig. 1 and Supplementary Fig. 1 for detailed results).

Sensitivity analyses reproduced the identified disease-associated degeneromes both vs. an expanded set of healthy controls including the lower age range (Supplementary Fig. 2), using the superdense normative connectome (Supplementary Fig. 3), and investigating DTI-derived fractional anisotropy (Supplementary Fig. 4).

### Z-value-based analysis of degeneromes on the single-patient level

**Fig. 2:**
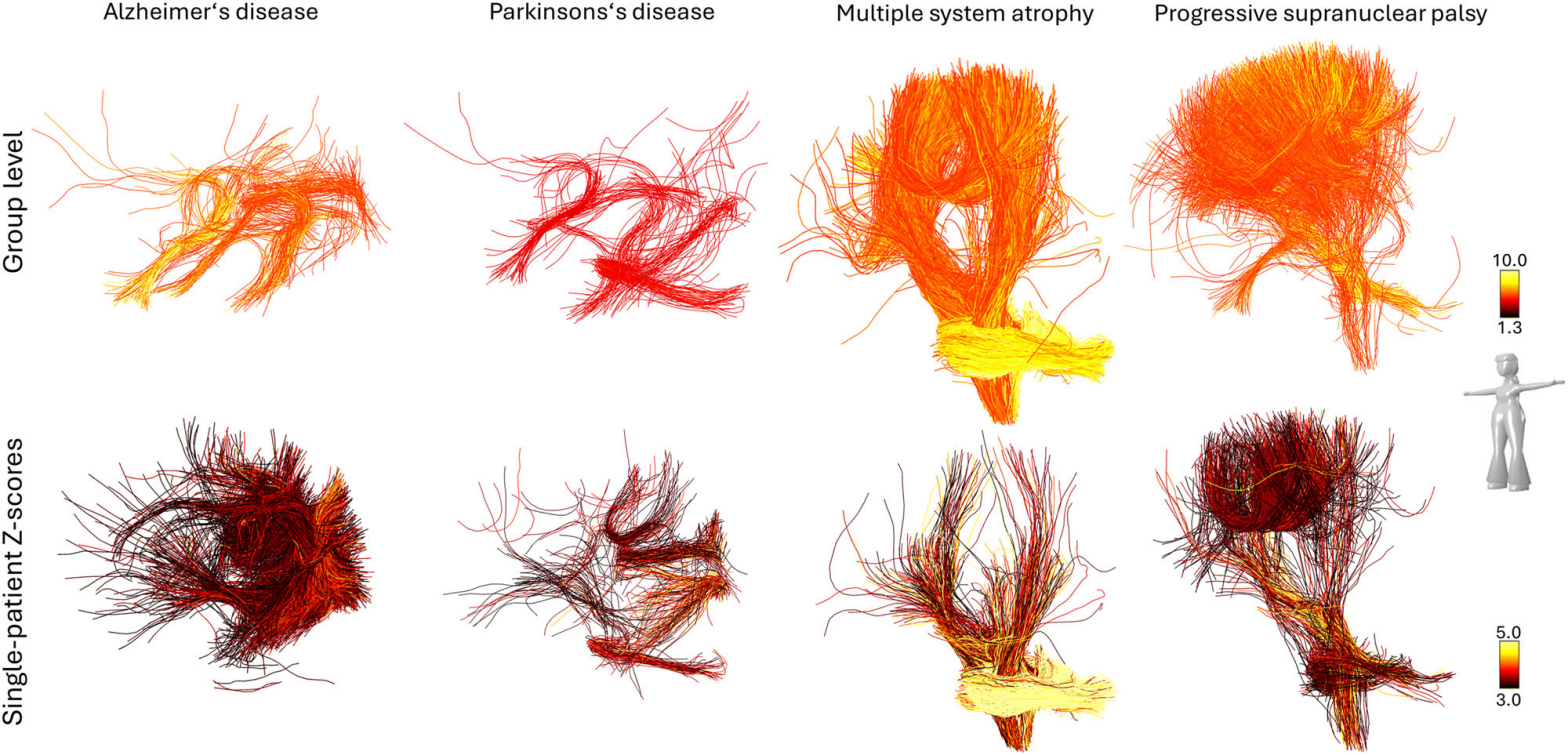
Comparison of group-level degeneromas (top row) and single-patient Z-score visualizations (bottom row) of abnormally reduced diffusion microstructure imaging-derived intraaxonal volume fraction (V-intra; z-scores <3.0).

Z-scores along normative connectome streamlines were successfully computed for the cohort. At the individual level, patients exhibited patterns of microstructural disruption that closely mirrored the group-level findings. Exemplary cases are shown in Fig. 2.

## Discussion

Here, we present a novel streamline-wise framework for the statistical analysis of brain imaging data that facilitates mapping axonal neurodegeneration along white-matter pathways and derives disease-specific fiber “degeneromes”. Applied to AD, PD, PSP, and MSA, the method yields distinct signatures that align closely with established pathoanatomical disease models and support robust group-level differentiation between entities. Beyond classification, it provides an atlas-independent, connectome-level view of network disruption. Finally, a z-score implementation enables intuitive single-patient visualizations, underscoring the translational potential of fiber degeneromes as clinically usable biomarkers—particularly for challenging differential diagnoses such as atypical Parkinson syndromes.

The disease-specific patterns of white-matter degeneration identified in the present study closely mirror established pathoanatomical and network-based models of neurodegeneration and are strongly supported by prior diffusion MRI evidence. In Alzheimer’s disease, white-matter disruption predominantly affects long-range limbic and posterior association pathways, particularly the cingulum bundle and related tracts linking medial temporal structures with the posterior cingulate cortex and precuneus, consistent with diffusion-based demonstrations of early cingulum and fornix involvement^25–27^. In Parkinson’s disease, alterations extend beyond nigrostriatal circuits to involve long-range occipito-temporal and fronto-occipital association pathways that support ventral visual and associative networks, in line with tract-specific diffusion abnormalities reported in the inferior longitudinal and inferior fronto-occipital fasciculi and their association with cognitive and visuospatial deficits^28–30^. In contrast, MSA and PSP are characterized by prominent degeneration of frontal–subcortical motor circuits and fronto-parietal control networks, reflecting their predominant motor and executive clinical phenotypes; diffusion MRI studies demonstrate marked involvement of corticospinal, fronto-striatal, and frontal association tracts in MSA^31–33^ and severe disruption of frontal white matter, including the superior longitudinal fasciculus and callosal fibers, in PSP^34–36^. Taken together, these convergent findings support the concept of a disease-specific white-matter “degenerome,” whereby selective vulnerability of large-scale brain networks underlies the distinct clinical and anatomical signatures of neurodegenerative disorders, in line with network-based models of neurodegeneration^37^.

In the present study, we provide a proof-of-concept demonstration of the “degenerome” framework at the group level and outline its diagnostic potential through application at the single-patient level. Future work should extend this approach to clinically meaningful outcomes by linking degenerome patterns to symptoms and established clinical metrics across neurodegenerative diseases. A useful conceptual parallel comes from stroke research, where domain-specific cognitive deficits often arise from disruption of wide-spanning networks rather than damage to a single cortical locus. “Disconnectome” methods operationalize this principle by estimating which white-matter pathways are interrupted and which remote regions are consequently disconnected, providing a mechanistically grounded measure of network-level impact^38^. In predictive modeling, structural disconnection maps explain granular cognitive outcomes at least as well as lesion topography alone^39^ and show strong performance for executive function and processing speed^40^. Extending this logic to neurodegeneration suggests that regionally selective pathology and axonal injury affect cognition not only through local gray matter loss, but also via progressive disconnection of long-range white matter pathways. This motivates a shift from lesion-derived disconnectomes to degeneration-derived “degeneromes”—connectome-informed maps that quantify pathway-specific vulnerability and the distributed network footprint of neurodegenerative processes. Consistent with this framework, altered white matter connectivity has been associated with early cognitive impairment in AD and PD^7–9^.

Most neurodegenerative diseases are proteinopathies, defined by the accumulation and spread of misfolded proteins. These pathological species propagate in a regionally selective manner and may follow anatomical connectivity, including transmission along fiber pathways and trans-synaptic routes^41^. In this context, white matter constitutes a structural scaffold that can both constrain—and potentially facilitate—the spatial distribution of pathology and is increasingly recognized as a key substrate of neurodegenerative change. In α-synucleinopathies, network-based propagation is a well-established concept^42,43^. A similar connectivity-driven mechanism has also been proposed for tauopathies. Converging preclinical evidence indicates that pathological tau can spread cell-to-cell along neuronal connections, consistent with prion-like behavior^44–46^. In mouse models, intracerebral delivery of tau seeds leads to preferential spread into anatomically connected regions, supporting circuit-based propagation rather than passive diffusion into spatially adjacent tissue^27^. Mechanistically, synapses and neuronal activity appear to facilitate this process: neuronal activity stimulates tau release, synaptic contacts enhance intercellular transfer, and optogenetic activation accelerates tau propagation in vivo^47,48^. Taken together, these findings implicate neuronal connectivity as a major route for tau dissemination. Consistent with this framework, multimodal imaging that combines tau-PET with resting-state fMRI has linked connectome organization to the topology of tau deposition in AD^49^ and PSP^50^. Extending this concept, recent in-vivo multimodal imaging suggests that tau (and associated neuroinflammatory signals) aligns with principal gradients of functional and structural brain organization in AD, further supporting a strong link between connectome architecture and spatial patterns of pathology^51^. Collectively, these observations highlight white matter and structural connectivity as central factors in the propagation and pathogenesis of neurodegenerative disease. Against this background, representing a “degenerome” as the already compromised component of a network may provide mechanistic insight into disease progression and, importantly, enable prognostic inferences by capturing the distributed footprint of ongoing neurodegeneration.

Technically, the proposed streamline-wise framework offers several advantages over voxel-/fixel-based inference. By operating on tract- and streamline-defined units, it reduces the dimensionality of the inference problem (i.e., fewer statistical tests than voxel-wise mapping) while preserving biologically meaningful organization along axonal trajectories^22^. This topology-aware representation has intrinsic spatial coherence, which makes it less dependent on cluster-enhancement heuristics that are commonly used for voxel-wise statistical maps (TFCE)^52^. Importantly, the resulting readouts are pathway-level and therefore directly interpretable and communicable as tract- or network-specific signatures. The method is modality-agnostic in the sense that streamline-/tract-wise sampling can be applied to quantitative scalar maps once they are brought into a common space, and related streamline-wise/connectome-based inference frameworks have been demonstrated for diffusion-derived microstructural measures^20^. Moreover, using a normative connectome in standard (MNI) space provides a practical route to tract/streamline-level interrogation without requiring subject-specific tractography for every application^53^. Finally, normative-reference approaches at the streamline/pathway level enable interpretable single-subject assessments (e.g., z-scores/percentile-based deviations from a norm). This might be employed for individualized profiling and potential longitudinal monitoring, though this requires validation in future research.

This study has several limitations as it is a retrospective, single-center, cross-sectional analysis, which limits generalizability and precludes inferences on disease trajectories; in particular, clinical associations and longitudinal progression within each disease entity are beyond the scope of this work. Additionally, tract-level summarization using BCBtoolkit ROIs provides a pragmatic whole-brain reporting layer, but its tract atlas does not fully cover all white-matter pathways; while we are not aware of a clearly superior, comparably comprehensive whole-brain alternative for this purpose, incomplete tract allocation may lead to underrepresentation of certain bundles in ROI-based summaries. A methodological limitation is that each streamline was represented by one averaged value, which improves statistical tractability at whole-connectome scale but reduces along-tract specificity and may dilute focal abnormalities within long or geometrically complex pathways. Group-averaging of diffusion data and spatial normalization may reduce anatomical fidelity at the gray-white matter interface and in superficial or highly variable white matter. Finally, although the resulting degeneromes show strong biological plausibility and discriminatory potential, external validation in independent cohorts, as well as assessment of test-retest reliability and robustness across sites/scanners, is still pending.

In conclusion, streamline-wise mapping of axonal microstructure provides a connectome-informed, biologically grounded readout of neurodegeneration that complements local voxel-/fixel-based approaches and avoids reliance on predefined parcellations. Across AD, PD, MSA, and PSP, the resulting fiber “degeneromes” revealed distinct, tract-matched signatures that closely align with established pathoanatomical models and enabled robust group-level differentiation—particularly between atypical parkinsonian syndromes. By expressing degeneration along white-matter trajectories, the framework offers an intuitive, pathway-level view of distributed network disruption with direct clinical interpretability. Finally, normative streamline-wise z-scores translate these group signatures to the single-patient level, enabling straightforward visualization and laying the groundwork for individualized reporting and longitudinal monitoring.

## Methods

### Study participants and clinical outcomes

This retrospective, single-center, cross-sectional study included diffusion microstructure imaging (DMI) and T1-weighted MRI data from four patient groups: (I) 81 patients with Alzheimer’s disease (AD; mean age: 69.0 (standard deviation 6.7) years; median disease duration: 2.0 [interquartile range 2.0] years; 47 females), (II) 177 patients with Parkinson’s disease (PD; mean age: 64.0 (standard deviation 9.0) years; median disease duration: 8.0 [interquartile range 7.0] years; 64 females), (III) 50 patients with (MSA; mean age: 63.6 (standard deviation 9.0) years; median disease duration: 3.0 [interquartile range 3.75] years; 27 females), and (IV) 35 patients with (PSP; mean age: 72.7 (standard deviation 7.1) years; median disease duration: 2.0 [interquartile range 2.0] years; 18 females). MRI examinations were performed as part of routine clinical work-up for differential diagnosis and to guide optimization of treatment strategies (e.g., prior to deep brain stimulation) between January 2018 and March 2024. The study was approved by the Institutional Review Board of the University of Freiburg (Ethics Committee—University of Freiburg, EK 400/20) and conducted in accordance with the Declaration of Helsinki and its later amendments. Owing to the retrospective design, the requirement for written informed consent was waived. Neurodegenerative diagnoses were established according to current consensus criteria for AD, PD, MSA, and PSP^54–57^. Datasets with corrupted image data were excluded. Patients were compared with a healthy control (HC) group (*n* = 26; mean age: 65.0 (standard deviation 8.0) years; 14 females) without known neurological disease, without neurological deficits on clinical examination, and without a family history of neurodegenerative disease. In addition, HC participants showed no neuropsychological impairment according to the Montreal Cognitive Assessment.

Statistical analyses of demographic data were performed using R (https://www.R-project.org/) and SPSS version 25 (IBM, Ehningen, Germany). The Shapiro-Wilk test was used to assess the normal distribution of data. In the case of normal distribution, data were indicated as mean (standard deviation) and t-tests were used for group comparisons. If data were not normally distributed, data were indicated as median [interquartile range]. Categorial data was compared using χ²-test. Bonferroni correction was applied for multiple comparisons.

With respect to age, a significant difference was observed only between the HC and PSP groups (t-test, *p* < 0.001 after Bonferroni correction for multiple comparisons). With respect to sex, no significant differences were found between HC and any patient group (χ²-test, all *p* > 0.05).

### MRI acquisition and calculation of DMI parameters

MRI was performed with a 3 Tesla scanner (MAGNETOM Prisma, Siemens Healthcare, Erlangen, Germany) with a 64-channel head and neck coil. T1-weighted (T1w) images were acquired with a three-dimensional (3D) magnetization-prepared 180° radio-frequency pulses and rapid gradient-echo (MP-RAGE) sequence (repetition time: 2500 ms, echo time: 2.82 ms, flip angle: 7°, TI = 1100 ms, GRAPPA factor = 2, 1.0 mm^3^ isotropic voxels, 192 contiguous sagittal slices). The diffusion weighted sequence was acquired with the following parameters: axial orientation, 42 slices, voxel size 1.5 × 1.5 × 3 mm^3^, TR 2800 ms, TE 88 ms, bandwidth 1778 Hz/Px, flip angle 90°, simultaneous multi-band acceleration factor 2, GRAPPA factor 2, 58 diffusion-encoding gradient directions each with b-factors 1000 and 2000 s/mm^2^, 15 non-diffusion weighted images (interleaved during diffusion-encoding directions). The main acquisition used an anterior-to-posterior phase-encoding direction (A»P). In addition, a short reverse phase-encoding reference acquisition was obtained in the opposite posterior-to-anterior direction (P»A) with matched geometry and readout parameters (42 slices, 1.5 × 1.5 × 3 mm^3^, TR 2800 ms, TE 88 ms, bandwidth 1778 Hz/Px), comprising 12 diffusion directions with *b*-values of 0 and 500 s/mm^2^. For all groups, MRIs were acquired over the same period of time, with the same scanner, same coil and same sequence parameter settings.

Data processing was implemented within our in-house post-processing platform NORA (www.nora-imaging.org) and performed as previously described^58^. An overview is shown in Fig. 1. Pre-processing of diffusion-weighted images included denoising^59^, correction of Gibbs-ringing artifacts^60^, and upsampling to an isotropic resolution of 1.5 mm³. Upsampling was used to improve interpolation and streamline sampling on a common grid, but it does not increase the true acquired spatial resolution. Susceptibility-induced distortions were corrected using FSL topup based on pairs of images with reversed phase-encoding directions, enabling estimation of the underlying off-resonance field. We did not do any eddycurrent correction due to the bipolar diffusion gradient preparation. Microstructural diffusion metrics were then estimated using a Bayesian approach that models three components of a white-matter tissue standard model^13,61^: (i) the free-water/CSF fraction (V-CSF), in which molecules move freely over distances on the order of tenths of micrometers; (ii) the volume fraction within axons (V-intra) with near one-dimensional diffusion constrained by tight membrane boundaries; and (iii) the volume fraction outside axons and dendrites (V-extra), characterized by an intermediate level of diffusion restriction, representing the cellular compartment and extracellular matrix. T1-weighted (T1w) datasets were automatically segmented into white matter, gray matter, and cerebrospinal fluid using CAT12 (http://www.neuro.uni-jena.de/cat/), and the dMRI images were coregistered to the T1w images. The validity of coregistrations between dMRI images and T1w-derived tissue probability values (TPV) was confirmed by visual inspection. Additional quality control included visual review of each individual DMI map and CAT12 segmentation. No dataset was excluded due to this.

### Normative connectome generation

We constructed a normative structural connectome using diffusion MRI data from the Human Connectome Project (HCP). Individual diffusion-weighted datasets were spatially normalized to a common MNI reference space using the nonlinear warp fields provided by the HCP preprocessing pipeline. During spatial normalization, local Jacobian information was taken into account to appropriately reorient diffusion gradient directions, thereby preserving the physical validity of the diffusion signal under deformation. Following normalization, diffusion data were averaged across subjects in group space to obtain a high signal-to-noise normative diffusion dataset. For subsequent modeling, the diffusion signal was represented on a 128-direction shell (as opposed to the 91 directions used in the original HCP acquisition) to ensure a sufficiently dense and homogeneous angular sampling for tractography. This representation was obtained via spherical interpolation of the diffusion signal using trilinear interpolation based on barycentric coordinates on a tessellated sphere, allowing consistent resampling of all subjects onto a common set of gradient directions. Whole-brain global tractography was then performed on this group-averaged diffusion volume using the global tracking toolbox^62^ (implementation available at https://bitbucket.org/reisert/globaltracking), applying both the “sparse” and “dense” reconstruction presets. In contrast to conventional local streamline approaches, global tractography does not rely on fiber orientation distribution reconstruction or explicit seeding strategies. Instead, it formulates tractography as a global optimization problem, where the complete set of streamlines is iteratively adapted to best explain the measured diffusion signal under a generative model. This framework inherently enforces spatial coherence and provides robustness to noise, partial volume effects, and moderate smoothing introduced by averaging. For the analyses presented here, the sparse reconstruction was used, resulting in a normative connectome comprising approximately 20,000 streamlines. The resulting normative tractogram is made publicly available for download [https://bitbucket.org/reisert/degenerome]. In addition to the “sparse” reconstruction used in the present analyses, we also provide alternative, denser (“dense”) reconstructions with approximately 163,000 and 339,000 streamlines for applications requiring higher streamline sampling density.

### Spatial normalization and voxel/region-wise comparisons

**Fig. 3:**
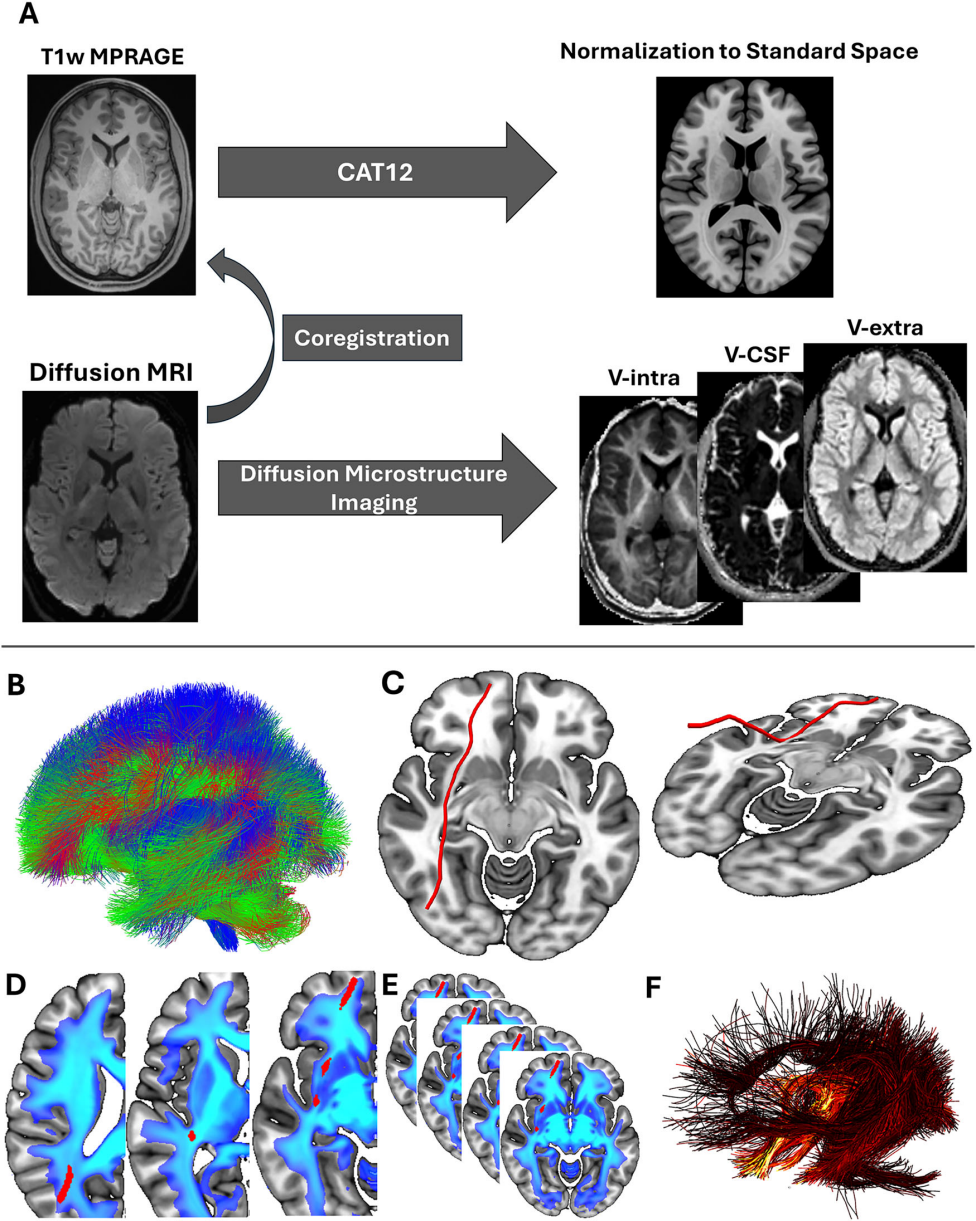
Overview of the workflow and developed analysis method. **A** Structural T1-weighted MPRAGE data is normalized to standard space using CAT12. Multishell diffusion-weighted imaging is coregistered to T1-weighted data and processed using diffusion microstructure imaging to obtain voxel-wise information of the volume fractions of the free fluid (V-CSF), the intraaxonal (V-intra) and the extraaxonal cellular and extracellular compartment (V-extra). **B** In standard space, a normative connectome is employed. **C** Individual patient data is warped to standard space. Here, for each streamline (one exemplarily depicted in red), imaging parameter information is collected along the streamline trajectory (a fiber visit map is given in red and overlaid onto a quantitative MRI map (**D**)). This is carried out across the whole cohort and all streamlines of the normative connectome (**E**), allowing for conventional statistical analysis across the whole connectome (**F**).

Our approach is visualized in Fig. 3. To display white matter fiber integrity, we focused on V-intra, as the demise in the axonal microstructural compartment was shown to be the most sensitive measure of fiber damage in multiple sclerosis and cervical spinal stenosis^63,64^. For gray matter, V-intra can be interpreted as the compartment corresponding to dendritic branches or glial processes^65^. To investigate the spatial distribution of V-intra decrease by voxel-wise analysis, images were spatially normalized by CAT12 and the diffeomorphic anatomical registration using the exponentiated lie algebra (DARTEL) method^66^. The diffeomorphic warp was used to transfer the quantitative dMRI maps to the Montreal Neurological Institute (MNI) space. Images were smoothed with a 3 mm full-width at half-maximum (FWHM) Gaussian kernel. As implemented in the Statistical Parametric Mapping-Voxel-Based Morphometry (SPM-VBM) 8-Toolbox, voxel-based group comparison between a patient group (i.e., AD, PD, MSA or PSP) and controls of the whole-brain DMI parameters was performed using a parametric multiple regression model, whereas the parameters “age” and “sex” served as covariates. The false discovery rate (FDR)-method was employed to correct for multiple comparisons.

### Streamline-wise comparisons and mapping of affected white-matter tracts

To further investigate the white-matter networks that were particularly affected by neurodegeneration-related V-intra changes, we used the normative structural connectome in MNI space. Individual V-intra maps were first warped from subject space into MNI space as described above. For each streamline, the V-intra values of all voxels intersected by that streamline were then averaged to obtain a streamline-specific value. Subsequently, whole-connectome, streamline-wise comparisons between patients and healthy controls were performed using a parametric multiple regression model with “age” and “sex” as nuisance covariates. Streamlines showing significant group differences after False discovery rate (FDR) correction to account for multiple comparisons were visualized to depict the “degenerome”. We employed the BCBtoolkit (http://toolkit.bcblab.com/) to identify which major white-matter tracts were most affected by degeneration. White-matter tract regions of interest (ROIs) provided within the toolbox were used. For each group and each ROI, we quantified the total length of all degenerome streamlines intersecting that ROI and expressed this value in per mille (‰) of the streamline length of the total connectome intersecting that ROI.

Sensitivity analyses were carried out by carrying out the aforementioned streamline-wise group comparisons of the respective disease groups vs. healthy controls for (a) an expanded cohort of healthy controls including more individuals in the lower age range (*n* = 97, mean age of 42.2 years, 17.2 years standard deviation, range: 18–78 years; 49 females, 48 males), (b) versus the age-matched *n* = 26 healthy controls but using the “superdense” normative connectome instead of the “sparse” preset, and (c) comparing DTI-derived fractional anisotropy instead of V-intra as quantitative MRI metric of interest.

### Streamline-wise Z-value-based analysis on the single-patient level

To assess the potential of whole-brain connectome analysis in the circumstance of neurodegeneration, we developed a pipeline that allows for analysis on the single-patient level. For this, an age- and sex-matched (±10 years) sample of healthy controls is chosen for a patient. The aforementioned readout of quantitative DMI metrics along each streamline was implemented, and normative values per streamline were obtained from the control cohort. This facilitates the identification of streamline-based abnormal z-values for each quantitative parameter.

### Statistical analysis

Statistical analyses were performed using R (https://www.R-project.org/) and SPSS version 25 (IBM, Ehningen, Germany). The Shapiro–Wilk test was used to assess normal distribution of data. In case of normal distribution, data were indicated as mean (standard deviation) and t-tests were used for group comparisons. If data were not normally distributed, data were indicated as median [interquartile range] and nonparametric Mann-Whitney U-tests were applied. Bonferroni correction was applied for multiple comparisons.

## Supplementary information

Supplementary information

## Data Availability

Data is available from the authors upon reasonable request and approval of the ethics committee.

## Code availability

DMI parameter calculation was performed using the platform NORA (http://www.nora-imaging.org), with additional analyses based on CAT12/SPM-VBM and the publicly available global tracking toolbox (https://bitbucket.org/reisert/globaltracking). The normative tractogram used in this study, including sparse and dense reconstructions, is available at (https://bitbucket.org/reisert/degenerome).
