# Supplementary material for: The degenerome—a novel streamline-wise approach for white matter integrity in neurodegeneration
Source: NPJ Parkinsons Dis. 2026 Jun 10;12:139. doi: 10.1038/s41531-026-01428-2 (PMC13254335; doi:10.1038/s41531-026-01428-2)
Supplement: Supplementary file 1 — Supplementary information [file 41531_2026_1428_MOESM1_ESM.docx]

**Supplement**

# **
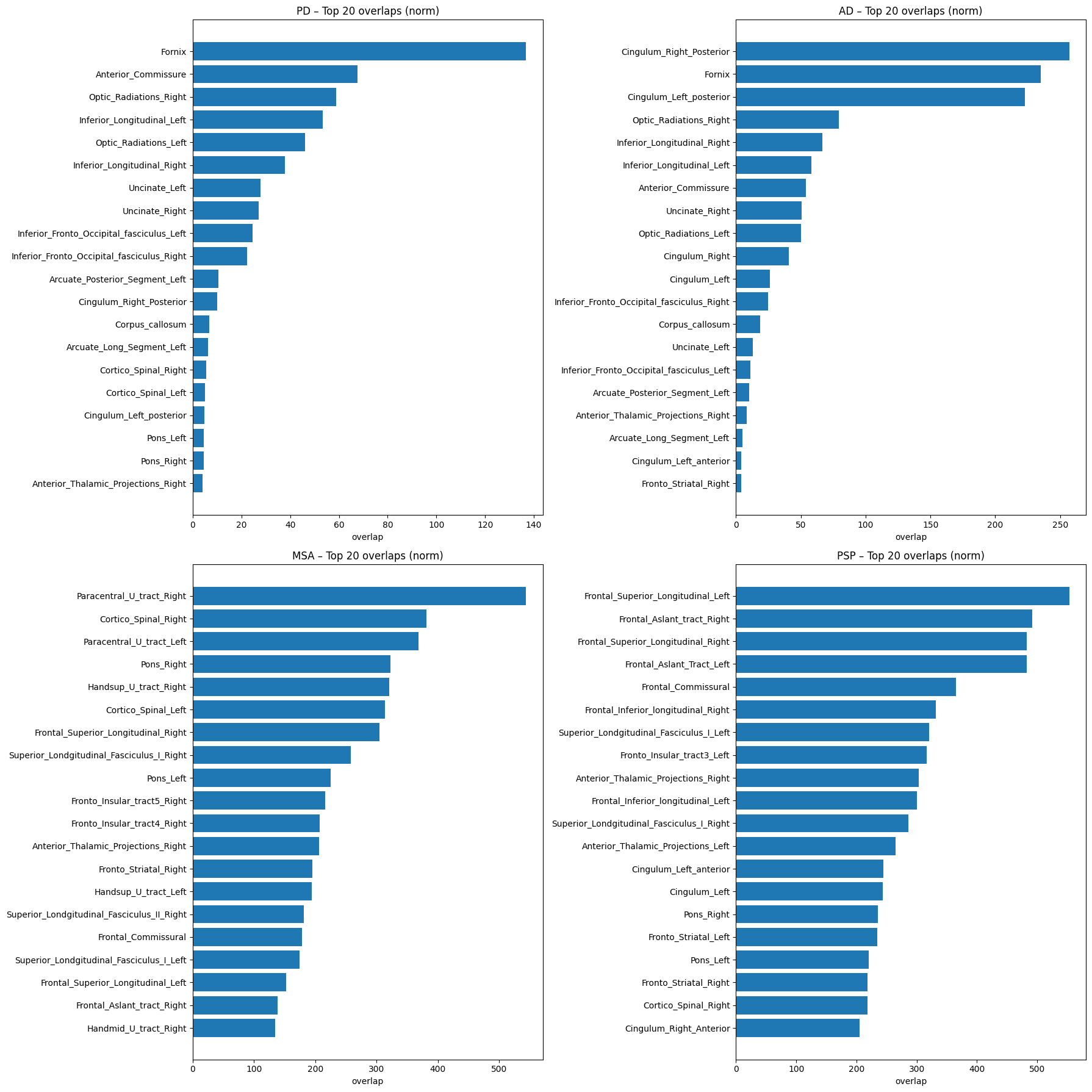
**

**Supplementary Figure 1 | Tract-specific white matter involvement across neurodegenerative diseases.**Bar plots depict the 20 white matter tract ROIs (BCBtoolkit) with the greatest overlap with disease-specific degenerome streamlines for Alzheimer's disease (AD), Parkinson's disease (PD), multiple system atrophy (MSA), and progressive supranuclear palsy (PSP). Degenerome streamlines were identified as those exhibiting significantly reduced DMI-derived intra-axonal volume fraction (V-intra) in patients relative to healthy controls, derived from a whole-connectome streamline-wise regression model (covariates: age, sex; FDR-corrected). Tract involvement was quantified as the cumulative length of significant degenerome streamlines intersecting each ROI, normalized to the total streamline length of the normative connectome within that ROI — yielding a normalized overlap score in which higher values reflect greater relative tract degeneration.


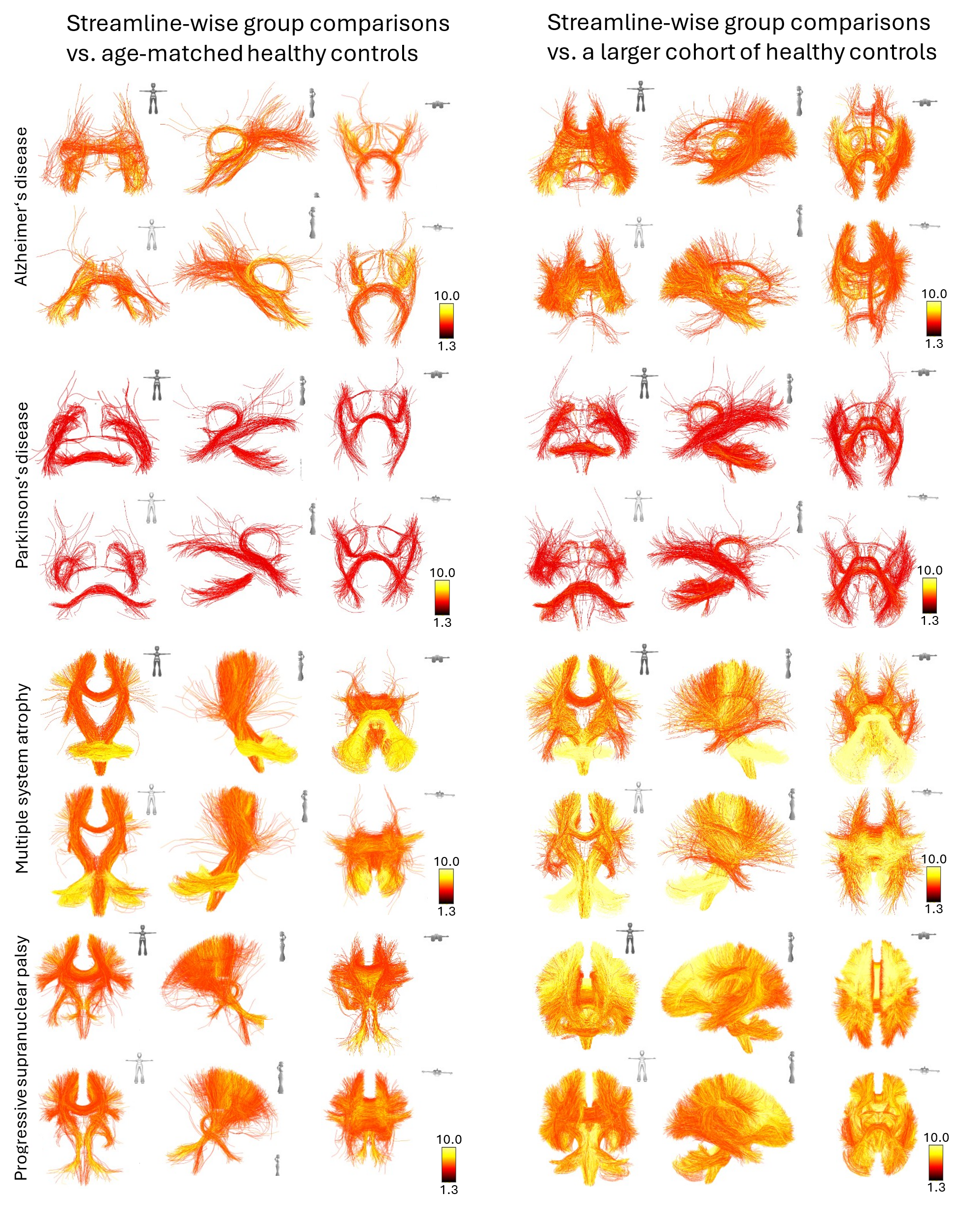


**Supplementary Figure 2 |** Comparison of the streamline-wise results of group comparisons adjusted for age and sex for V-intra of the studied disease groups vs. an age-matched cohort of healthy controls (n=26; left column) and an expanded cohort including healthy controls in the younger age range (n=97, mean age of 42.2 years, 17.2 years standard deviation, range: 18-78 years; 49 females, 48 males).


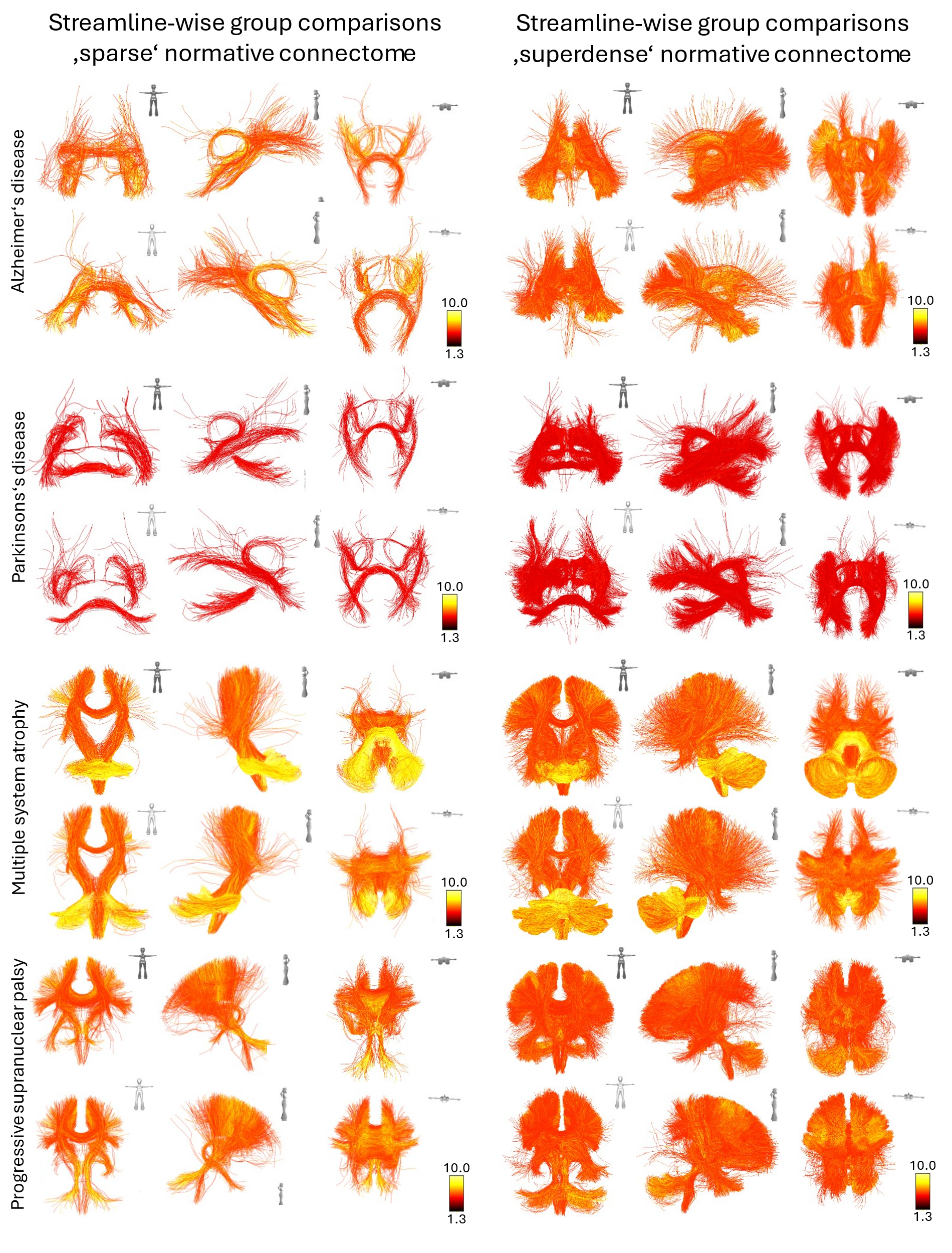


**Supplementary Figure 3 |** Comparison of the streamline-wise results of group comparisons adjusted for age and sex for V-intra of the studied disease groups using the “sparse” (left column) and “superdense” (right column) setting of the normative connectome.

**
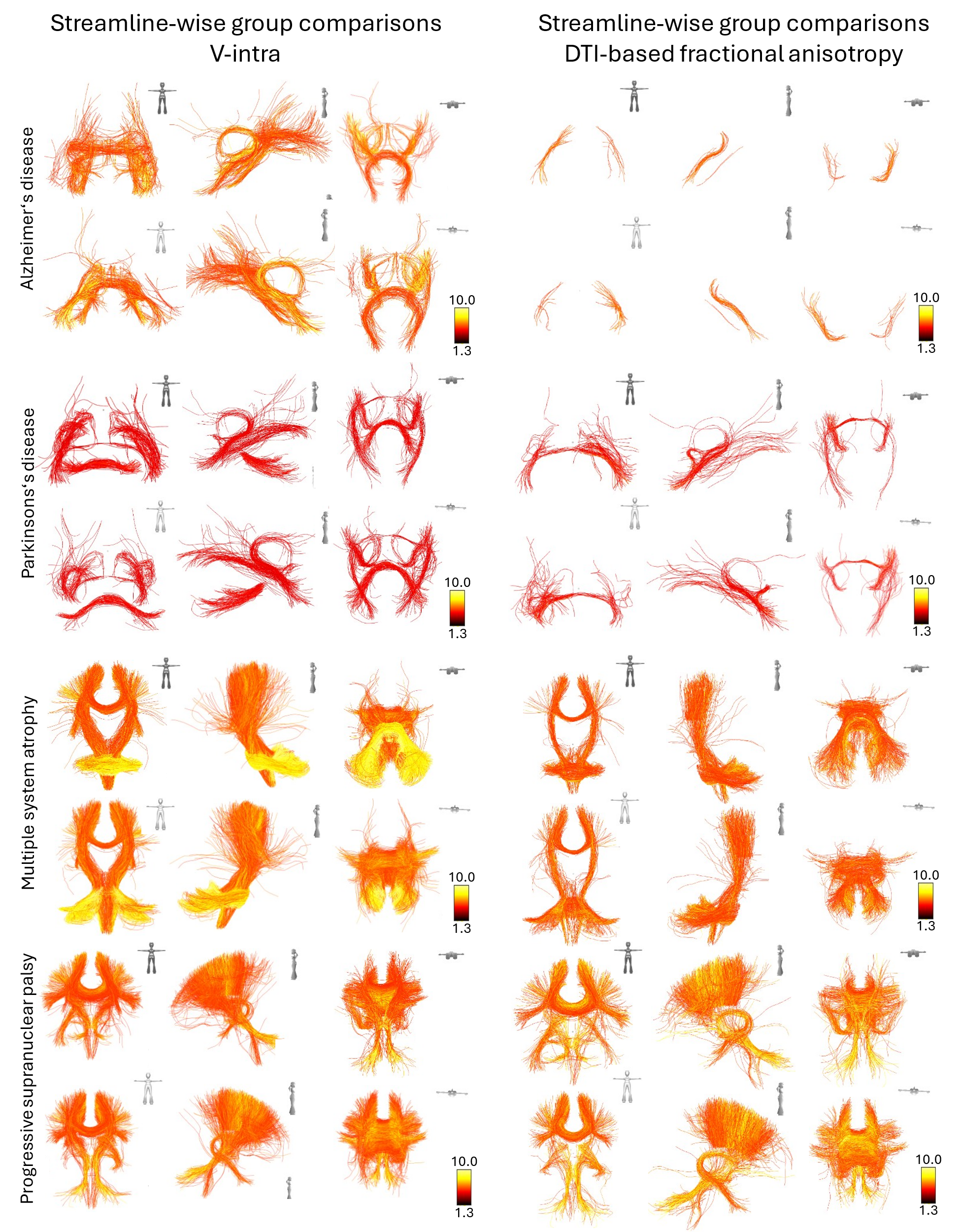
Supplementary Figure 4 |** Comparison of the streamline-wise results of group comparisons adjusted for age and sex for V-intra (left column) and DTI-derived fractional anisotropy (right column).
